# Income inequality among general practitioners in Iran: a decomposition approach

**DOI:** 10.1186/s12913-019-4473-7

**Published:** 2019-09-02

**Authors:** Mohsen Bayati, Arash Rashidian, Yaser Sarikhani, Saeed Lohivash

**Affiliations:** 1 Health Human Resources Research Center, School of Management & Information Sciences, Shiraz University of Medical Sciences, Shiraz, Iran; 2 Department of Health Management and Economics, School of Public Health, Tehran University of Medical Science, Tehran, Iran; 3 Director of Information, Evidence and Research, Eastern Mediterranean Region, World Health Organization, Cairo, Egypt; 4 Student Research Committee, School of Management & Information Sciences, Shiraz University of Medical Sciences, Almas Building, Alley 29, Qasrodasht Ave, Shiraz, Iran

**Keywords:** Income gap, Income inequality, General practitioner, Remuneration

## Abstract

**Background:**

General practitioners (GPs) are among the most important resources of healthcare system and public health is considerably influenced by the function of this group. Income inequality among GPs considerably affects the motivation and performance of this group. The present study aims to examine the income inequality among Iranian GPs in order to provide the necessary evidence for health human resource policy.

**Methods:**

In this cross-sectional study, the distribution of income and wage inequality among GPs was investigated using income quintiles. We also used the Dagum’s model to analyze the inequality between different groups of GPs through the decomposition of the Gini coefficient. Moreover, a regression model was used to determine the effective factors on GPs’ income.

**Results:**

The results of this study indicated that income and wages of GPs in the highest quintile were eight times more than those of doctors at the lowest quintile. Regression estimates showed that factors such as gender, practice setting, and activity as the family physician (*P* < 0.001) were effective on income of GPs; and also male and self-employed GPs had significantly more wage (*P* < 0.001). Total Gini coefficient of GPs’ income and wage were estimated at 0.403 and 0.412, respectively. Highest monthly income was found in GPs with 16–20 years practice experience ($8358) based on Purchasing Power Parity (PPP), male ($8339 PPP), and self-employed GPs ($8134 PPP) subgroup. However, the female ($5389 PPP) and single ($5438 PPP) GPs had the lowest income. Population share; income/wage share; income/wage mean; Gini coefficient; and within, between and overlap decomposed components of Gini coefficient are also reported for each GPs subgroups.

**Conclusions:**

We found significant inequalities in income and wages among Iranian GPs. Adjustment of income based on working hours indicated that one of the most common causes of income inequality among GPs in Iran was different workloads among different groups. Since the motivation and function of physicians can be influenced by income inequality, policymakers in the health system should consider factors increasing such inequalities.

**Electronic supplementary material:**

The online version of this article (10.1186/s12913-019-4473-7) contains supplementary material, which is available to authorized users.

## Background

Health professionals and especially General practitioners (GPs) are among the most important resources of health systems [1]. Performance of health systems is deeply dependent on GPs, so that effectiveness of health systems in achieving their goals is to a large extent subject to adequate, well-qualified, and motivated GPs [2]. Financial incentives are among the most important determinants of GPs’ behavior and performance. Quality and quantity of health services, total health system costs, motivation of GPs, and retaining them in a health system are profoundly correlated with financial incentives and especially income equality [3, 4].

Inequality in GPs’ payments and incomes will lead to undesirable consequences [5]. Income inequality can provoke GPs to reduce quantity or quality of services which can threaten public health [3]. Perception of income inequality by GPs may result in request of informal payments [6, 7] and this kind of out-of-pocket payment reduces access to health care and increases total health costs [8]. Inter-sectional income inequalities could encourage GPs to opt out of public health sector or health insurance schemes [9], or may increase rate of dual practice and it will restrict capacity of service delivery in public health section [10, 11].

Income inequality among GPs in different geographic regions of a country would lead to inland migration and eventually will worsen the distributional imbalance of GPs and constrain access to health services in underprivileged areas [10, 12]. Perception of Income gap by GPs may act as an incentive for general and primary care physicians to enter specialty training. Consequently, this will result in increased health expenditures and shortage of GPs in primary health care [13, 14]. Finally, income inequality may convince GPs to migrate to other countries looking for better financial opportunities [15].

There are few research studies on GPs’ income inequality which have mostly studied determinants of income gap across different groups. A study in Australia showed that GPs who worked in outer regions or rural areas and those who worked in personal office had higher income [16]. Another study in France demonstrated that male GPs had higher income than females, this result was explained with respect to different workloads [17].

In Iran, we also found a study by Bayati et al., which describe the economic behavior of GPs in terms of practice income, hours of work, and patient visits across different GPs groups [18]. Based on our searches, no study has comprehensively investigated income inequality among Iranian GPs. Therefore, this study aimed to investigate income inequality among GPs in Iran using Gini coefficient decomposition method to provide a reliable evidence for health human resource policy and planning.

## Method

### Data and variables

In this empirical cross-sectional study a total number of 666 Iranian GPs were participated. The data were collected using a researcher-made checklist on the economic behavior of GPs. The checklist was developed based on literature review and several semi-structured interviews with GPs. Several variables such as demographic characteristics, practice related factors and income variables were asked in the checklist. The checklist is provided as Additional file 1. Due to incomplete and out of date directory of Iranian GPs especially on private sector GPs, data were collected through simple sampling method in two national conferences held in 2016 and 2017 with a response rate of 52%. These national conferences for Iranian GPs are held by The Iranian Society of General Practitioners several times in a year. The GPs throughout the country have to participate in the conferences for Continuous Medical Education (CME) which is required for physicians to be able to continue their practice license.

In the present study, inequality among GPs was investigated in terms of income and wage. Total amount of gross monthly earnings due to medical practice was considered as “income”. Considering that income is affected by the amount of activity, the “wage” (income per hour) was also analyzed. Income inequality was analyzed based on demographic and medical practice variables. Demographic variables included gender, age, and marital status. Medical practice variables included practice setting, working as family physician, place of practice and experience of medical practice.

In order to convert the Iranian Rial figures to the United States Dollar, we used the exchange rate of Central Bank of the Islamic Republic of Iran in the study year (2015). Wage and monthly income were analyzed based on Purchasing Power Parity (PPP).

### Analysis

In the first step of data analysis, in order to evaluate the distribution of income among GPs, we computed income and wages quintiles, as well as the ratio of highest quintile to the lowest quintile. In order to analyze the amount of income inequality more precisely, determinants of income and wage were investigated using regression model. In the regression model, income or wages were estimated as a function of demographic and medical practice variables. Gross monthly income and the wage were logarithmically entered into the model as dependent variables.

Then the Gini coefficient (GC) of income and wage was estimated and finally inequalities between different groups of GPs were decomposed using the “Dagum 1997” approach. According to this approach, the GC is decomposed into three components including within subgroups, between subgroups and transvariation between subgroups.

The approach supposes that a population of size N is divided into k subgroups of sizes *N*_*j*_ (j = I, 2, 3, ..., k). In this population, population share of subgroups and income share of subgroups are shown by P and S, respectively. In this population, the income inequality (GC) decomposition can be considered as follows:

$$ {G}_{within}=\sum \limits_{j=1}^k{G}_{jj}{P}_j{S}_j $$

$$ {G}_{net\  between}=\sum \limits_{j=2}^k\sum \limits_{h=1}^{j-1}{G}_{jh}\left({P}_j{S}_h+{P}_h{S}_j\right)\ {D}_{jh} $$

In these formula, *D*_*jh*_ can be considered as overlap index. It takes values from 0 to 1, where a value of 0 demonstrate a perfect overlap between j and h subgroups $\left(\overline{Y_j}=\overline{Y_h}\right)$ and a value of 1 which shows there is not any overlap between them.

$$ {G}_{transvariation}=\sum \limits_{j=2}^k\sum \limits_{h=1}^{j-1}{G}_{jh}\left({P}_j{S}_h+{P}_h{S}_j\right)\ \left(1-{D}_{jh}\right) $$

The between subgroups inequality decomposes into net between subgroups inequality and transvariation between subgroups.

$$ {G}_{between}={G}_{net\  between}+{G}_{transvariation}=\sum \limits_{j=2}^k\sum \limits_{h=1}^{j-1}{G}_{jh}\left({P}_j{S}_h+{P}_h{S}_j\right) $$

Finally, overall inequality in population can be seen as:

$$ {G}_{Overall}={G}_{between}+{G}_{within}=\sum \limits_{j=1}^k{G}_{jj}{P}_j{S}_j+\sum \limits_{j=2}^k\sum \limits_{h=1}^{j-1}{G}_{jh}\left({P}_j{S}_h+{P}_h{S}_j\right) $$

More than GC decomposition of income and wage in GPs’ subgroups, the Lorenz curves of income and wage for subgroups of GPs were drawn. Data analysis was performed using STATA version 14.2 SE 64 (Stata Corporation 2015).

## Results

A total number of 666 GPs were participated in this study. Descriptive statistics of demographic and practice variables for the sample are presented in Table 1.

**Table 1:** Descriptive statistics of demographic and practice variables for sample of Iranian GP (*n* = 666)

Variables (Number of Respondents)	Frequency	Percent
Gender (665)		
Male	358	53.83
Female	307	46.17
Age (651)		
26–35 years	156	23.96
36–45 years	198	30.41
46–55 years	230	35.33
≥ 56 years	67	10.29
Marital status (663)		
Single	123	18.55
Married	540	81.45
Practice setting (664)		
Salaried	307	46.23
Self-employed	357	53.77
Family physician (661)		
Yes	69	10.44
No	592	89.56
Practice location (581)		
Capital of the country	219	37.69
Province centers	151	25.99
Other cities, villages	211	36.32
Practice experience (640)		
0–5 years	167	26.09
6–10 years	101	15.78
11–15 years	126	19.69
16–20 years	141	22.03
≥ 21 years	105	16.41

Table 2 indicates the GPs’ income and wage quintiles and the ratio of fifth quintile to the first quintile. The results revealed an unequal distribution of income among GPs, so that the income of the highest quintile was 8.6 times higher than the first quintile. The condition was almost the same for the wage, as the earning of GPs in the first wage quintile was about $5 per hour, while the figure for the last quintile was more than $45 per hour.

**Table 2:** Quintiles of Iranian GPs by income and wage

Quintiles	Income		Wage	
	Percent of GPs	Mean (SD)	Percent of GPs	Mean (SD)
Lowest	22.13	590.23 (239.82)	20.31	5.36 (1.70)
Second	20.06	1179.08 (65.65)	19.87	9.58 (1.14)
Third	19.59	1773.50 (283.58)	19.87	14.34 (1.66)
Fourth	18.95	2574.30 (174.68)	20.09	21.47 (2.58)
Highest	19.27	5106.81 (1781.17)	19.87	45.36 (20.98)
Highest to Lowest ratio	ـــــــ	8.65 (7.42)	ــــــــ	8.46 (12.34)

Determinants of income and wage are presented in Table 3. Results of the regression show that male GPs, those who worked at personal office, GPs who practiced as family physician, and those who worked in rural areas and small cities had significantly higher income. In the wage model, the results were slightly different, such that some variables were not significant. However, male GPs and those who worked in personal office had higher level of wage.

**Table 3:** Determinants of GPs’ earning (income and wage) in Iran

Variables	L Gross Monthly Income		L Wage	
	β^*^	*P*-value	β	*P*-value
Constant	15.438	0.000	10.279	0.000
Gender				
(1 female, 0 male)	−.532	0.000	−.159	0.041
Age				
26–35 years	Ref			
36–45 years	−.018	0.892	.062	0.682
46–55 years	−.204	0.188	−.090	0.592
≥ 56 years	−.626	0.001	−.627	0.002
Marital status				
(1 married, 0 single)	.047	0.583	.152	0.171
Practice setting				
(1 self-employed, 0 other)	.357	0.000	.457	0.000
Family Physician				
(1 yes, 0 no)	.377	0.000	.120	0.366
Practice location				
Other cities, Villages	Ref			
Province centers	−.108	0.146	.042	0.651
Capital of the country	−.186	0.010	−.033	0.712
Practice experience				
0–5 years	Ref			
6–10 years	.123	0.388	.281	0.059
11–15 years	.177	0.202	−.042	0.791
16–20 years	.357	0.017	.157	0.346
≥ 21 years	.274	0.104	.063	0.729
Goodness of fit	F = 14.51 (0.000), R^2^ = 0.25		F = 6.1 (0.000), R^2^ = 0.17	

β*: Regression coefficients

Population share, income share, monthly income, wage (hourly income), within subgroups GC, and the results of GC decomposition in terms of demographic and medical practice variables are shown in Table 4. For example, according to population share and income share figures, although male GPs are 53.3 percentage of all GPs, they have 63.9 percentage of incomes.

**Table 4:** Decomposition of GPs’ income inequality by demographic and practice factors

Variables	Population Share^a^	Income Share^b^	Income Mean (PPP^c^)	Gini Coefficient	Gini Decomposition			
					Within	Between	Overlap	Overall
Gender								
Male	0.534	0.639	8339.10	0.349	0.192 (0.476)	0.106 (0.263)	0.104 (0.260)	0.403
Female	0.466	0.361	5389.94	0.434				
Overall	1	1		0.403				
Age								
26–35 years	0.255	0.204	5919.32	0.418	0.109 (0.271)	0.076 (0.190)	0.216 (0.538)	0.403
36–45 years	0.291	0.299	7161.36	0.378				
46–55 years	0.353	0.383	7738.72	0.392				
≥ 56 years	0.101	0.094	6439.79	0.426				
Overall	1	1		0.403				
Marital status								
Single	0.186	0.146	5438.25	0.379	0.288 (0.716)	0.044 (0.111)	0.069 (0.172)	0.403
Married	0.814	0.854	7304.91	0.404				
Over all	1	1		0.403				
Practice setting								
Salaried	0.442	0.346	5471.60	0.389	0.201 (0.498)	0.098 (0.244)	0.103 (0.256)	0.403
Self-employed	0.558	0.654	8134.69	0.388				
Overall	1	1		0.403				
Family Physician								
No	0.886	0.881	6915.29	0.414	0.327 (0.811)	0.010 (0.026)	0.065 (0.161)	0.403
Yes	0.114	0.109	7153.44	0.306				
Overall	1	1		0.403				
Practice location								
Capital of the country	0.372	0.414	6306.80	0.434	0.101 (0.250)	0.161 (0.399)	0.140 (0.349)	0.403
Province centers	0.289	0.235	6808.16	0.406				
Other cities, villages	0.339	0.351	7435.22	0.337				
Overall	1	1		0.403				
Practice experience								
0–5 years	0.266	0.216	5624.94	0.375	0.074 (0.184)	0.116 (0.287)	0.212 (0.527)	0.403
6–10 years	0.161	0.143	6659.05	0.463				
11–15 years	0.192	0.196	6860.18	0.358				
16–20 years	0.218	0.260	8358.44	0.370				
≥ 21 years	0.163	0.185	7670.13	0.403				
Overall	1	1		0.403				

^a^Contribution of each group to the whole population

^b^Each group’s share of total income

^c^Purchasing Power Parity

Total Gini coefficient of GPs’ income was estimated at 0.403. The Gini coefficient is also reported in all subgroups based on different variables. The results revealed that the greatest inequality in the subgroups was related to the GPs with the practice experience of 6–10 years (GC: 0.463) and the lowest was related to the family physician subgroup (GC: 0.306). Based on some variables such as marital status and working/not working as family physician, the greatest share of income inequality was related to within group inequality. However, based on other variables such as age group, place of practice, and practice experience, the highest income inequality share was related to the gross between group inequalities. Also, based on gender and practice setting, the share of between and within group income inequality was approximately equal.

Findings of the study show that the GC of wage (0.412) was higher than income GC. The greatest wage inequality was related to the GPs with the practice experience of 11–15 years (GC: 0.478) and the lowest was related to the family physician subgroup (GC: 0.335). Results of decomposition of wage GC with few differences were similar to the decomposition of income GC. Based on some variables such as marital status, working/not working as family physician, and practice setting, the greatest share of wage inequality was related to within group inequality. Based on other variables such as age group, place of practice, and practice experience, the highest wage inequality share was related to the gross between group inequalities. Also, based on gender, the share of between and within group wage inequality was equal. Results of wage inequality are shown in Table 5.

**Table 5:** Decomposition of GPs’ wage inequality by demographic and practice factors

Variables	Population Share^a^	Wage Share^b^	Wage Mean (PPP^c^)	Gini Coefficient	Gini Decomposition			
					Within	Between	Overlap	Overall
Gender								
Male	0.567	0.576	61.80	0.379	0.206 (0.501)	0.009 (0.023)	0.195 (0.475)	0.412
Female	0.433	0.424	59.84	0.450				
Overall	1	1		0.412				
Age								
26–35 years	0.196	0.192	53.45	0.419	0.118 (0.288)	0.108 (0.263)	0.184 (0.447)	0.412
36–45 years	0.290	0.354	72.25	0.439				
46–55 years	0.386	0.398	62.79	0.376				
≥ 56 years	0.148	0.076	39.57	0.357				
Overall	1	1		0.412				
Marital status								
Single	0.152	0.117	46.28	0.399	0.312 (0.758)	0.047 (0.115)	0.051 (0.125)	0.412
Married	0.848	0.883	63.56	0.410				
Over all	1	1		0.412				
Practice setting								
Salaried	0.311	0.238	46.32	0.398	0.239 (0.580)	0.094 (0.231)	0.078 (0.189)	0.412
Self-employed	0.689	0.762	67.75	0.401				
Overall	1	1		0.412				
Family Physician								
No	0.902	0.920	62.33	0.417	0.347 (0.843)	0.027 (0.066)	0.037 (0.089)	0.412
Yes	0.098	0.080	49.24	0.335				
Overall	1	1		0.412				
Practice location								
Capital of the country	0.387	0.333	58.26	0.397	0.103 (0.251)	0.152 (0.368)	0.156 (0.379)	0.412
Province centers	0.279	0.275	65.54	0.424				
Other cities, villages	0.354	0.392	57.07	0.383				
Overall	1	1		0.412				
Practice experience								
0–5 years	0.211	0.189	51.51	0.402	0.079)0.192(	0.105)0.254 (	0.227)0.552(	0.412
6–10 years	0.147	0.181	71.74	0.391				
11–15 years	0.196	0.209	64.97	0.478				
16–20 years	0.255	0.279	64.21	0.367				
≥ 21 years	0.191	0.172	52.23	0.375				
Overall	1	1		0.412				

^a^Contribution of each group to the whole population

^b^Each group’s share of total wage

^c^Purchasing Power Parity

**Fig. 1:**
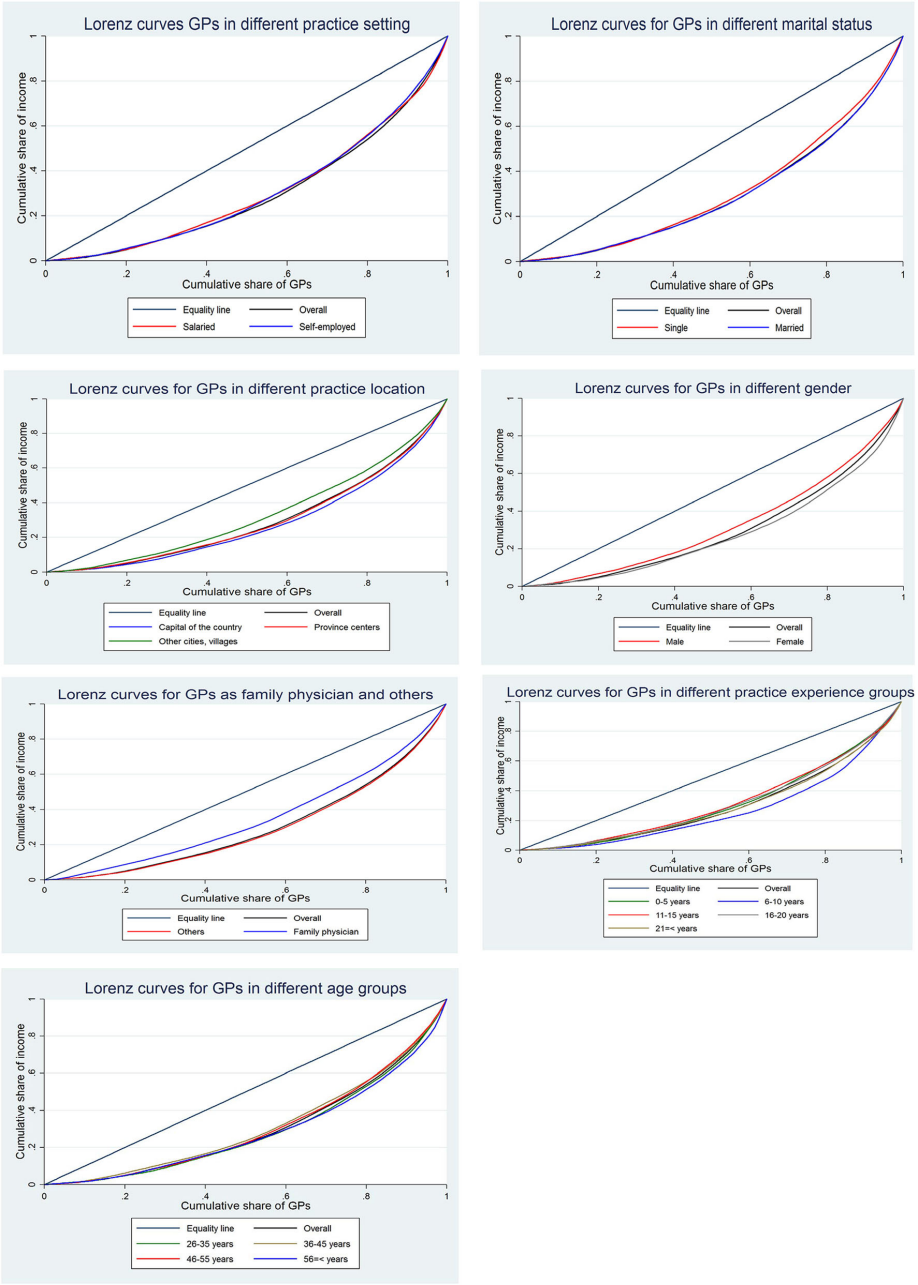
Lorenz curves for income distribution among different groups of Iranian GPs

**Fig. 2:**
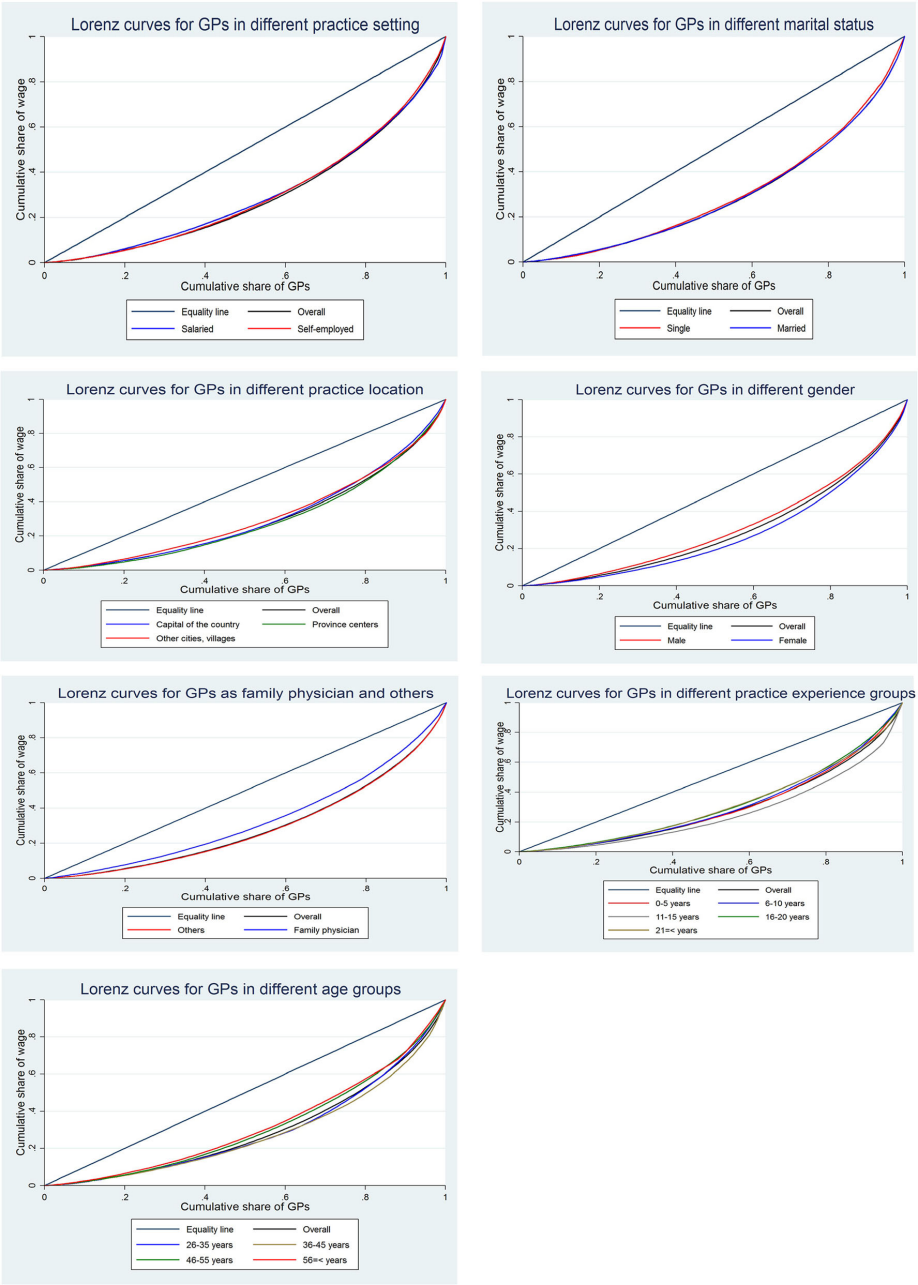
Lorenz curves for wage distribution among different groups of Iranian GPs

Figures 1 and 2 show the inequality of income and wage between different subgroups of GPs using Lorenz curves.

## Discussion

For the first time, this study has empirically investigated the income inequality among Iranian GPs. In the present study, in addition to income, GPs’ wage (income adjusted to working hours) was also studied to better interpret the inequality. However, in most of the studies conducted in this regard, gender inequality has been studied, the present article investigated income inequality in different groups based on gender, age, marital status, practice setting, place of practice and medical practice experience. In addition, in this study, appropriate methodology was used to analyze inequality. Dagum’s method in addition to decomposition of inequality into the between group and within group inequality components, considered the overlap of the groups and is therefore more accurate than other methods. Regarding the high amount of variables studied in this study, as well as due to insignificant effect of some variables on income and wage inequality among doctors, variables that were the main determinants of income in the regression model, were discussed.

The descriptive findings and the total Gini coefficient resulting from this study indicate significant inequalities in income and wages among GPs. The income and wages of physicians in the highest quintile were eight times more than the income and wage of physicians in the first quintile. Moreover, the Gini coefficient for GPs was 0.403, indicating high incomes and wage inequalities among Iranian physicians. Meanwhile, according to (United Nations Development Programme) UNDP, the Gini coefficient of income for the entire Iranian society was 0.383 in 2015 [19]. In line with the findings of the present study, the results of Homaie Rad et al. (2012) in Iran showed that the Gini coefficient of income for healthcare personnel in Iran was 0.428, and the Gini coefficient of the income for the whole society was 0.380 [5]. These findings reflect the fact that income inequality among GPs in Iran is more than income inequality in society and less than healthcare workers’ income inequality. Also results of a study among US physicians showed that Gini coefficient of income among physician during different years was between 0.3 to 0.33 for females and between 0.5 to .05 for males which are lower than Gini coefficient of income among Iranian Physicians [20].

Considering the fact that the gross monthly income is affected by the working hours of physicians, in this study, Gini coefficient of wage (monthly income in relation to working hours) is also estimated. The findings indicated that, despite the adjustment of income in terms of working hours, inequality was increased, and the Gini coefficient of wage was equal to 0.412. This suggests that other factors than working hours affect the income inequality of GPs in Iran. Along with the results of this study, the study conducted by Lin et al. in Taiwan showed that the number of working hours per week did not correlate with the amount of physicians’ income [21]. This is in line with the results of some studies which suggest a relationship between physicians’ income inequality and their working hours [14, 16]. In interpreting the relationship between the number of working hours and income, it is necessary to consider the type of service (outpatient and hospital) and the severity of the disease [14].

The results of this study showed that the Gini coefficient of income for male physicians was approximately 0.35 and it was 0.43 for female physicians, suggesting more income inequality among female doctors. From the total income inequality of GPs, 47% was related to the between group inequality. In addition, almost a quarter of the share of inequality in income was related to inequality between men and women. As showed in the Table 4, monthly mean income of male GPs was $8339 (PPP), however it was about $5390 (PPP) for female GPs. This gap between female and male physician’s earnings is common in other countries and it seems related to women preferences, financial expectation and limitations for job and earning [16, 22–24].

About 26% was related to the overlap between the two groups, which cannot be interpreted fully. On the other hand, in terms of wage inequality, the Gini coefficient for wages was 0.38 for men and 0.45 for women. The results of the decomposition of the Gini coefficient of wage indicated that more than half of the inequality in wages was within the groups, and about half the other is related to the overlap between groups, and the contribution of between group inequality is negligible. These findings can be indicative of the fact that although men have more income, but if the income is adjusted to the number of working hours, there is slight inequality among female and male physicians.

Homaie Rad et al. without mentioning the reasons for income inequality among different gender groups, pointed out that there was a significant difference between the income of women and men employed in the health sector of Iran [5]. Several studies, which are consistent with the results of the present study, have identified the main cause of income inequality among male and female GPs in their working hours [16, 17, 25, 26]. Nevertheless, the results of Duckett’s study in the United States, which contradict the interpretation of the results of the present study, showed that, apart from other factors such as working hours, there is a significant difference between the income of female and male employees in the health sector. He points out that the main reason for this income gap is the existence of gender discrimination [27].

The results of this study indicated that the Gini coefficient of income of self-employed physicians with a Gini coefficient of salaried physicians was approximately equal. The results of the decomposition of Gini coefficient revealed that the largest share of inequality was due to within group inequality (more than half of inequality). Subsequently, the major contributor to inequality was the overlapping of groups, and the share of income inequality between self-employed physicians and other physicals was also negligible. In terms of wage, the Gini coefficient of self-employed physicians and salaried physicians was approximately equal. The major share of wage inequality was related to within group inequality (58%) and about a quarter of it was related to between group inequality. Comparison of these results shows that when wage is investigated, the share of within group inequality increases significantly. This suggests that if income is adjusted to working hours, inequalities within self-employed doctors and other physicians are increased considerably.

According to present study, monthly revenue of salaried and self-employed GPs in Iran was about $5471 and $8134 (PPP), respectively. Their wage was also $46 and $67 (PPP), respectively. Morris’s study showed that self-employed GPs’ gross income was higher than those of other doctors employed in an organization. This study considered the main cause of income inequality as the difference in working hours of physicians in both groups [25]. Moreover, Cheng et al. by referring to the difference in income between self-employed physicians and other doctors, stated that the main reasons for this inequality were the difference in payment method and type of contract with insurance organizations [16]. In addition, other researchers mentioned that self-employed physicians due to more freedom in their practice and work usually have ability to make more money [16, 28].

The findings of this study showed that GPs working in family physicians had more income than other doctors. However, there was no significant difference between the wage of family physicians and other doctors by adjusting the income to working hours. These results may indicate that the main reason for the increased income of family physicians is their workload.

Although there was no significant difference between Gini coefficient of income and wage among different age groups of GPs in Iran, GPs over 56 years of age had higher Gini coefficient. These findings are in line with the results of a study among registered pharmacists in South Florida, USA. It is suggested that this inequality is related to the preferences of people of this age group for part-time working [29].

## Conclusion

This study showed that factors such as gender, work status, as well as activity as a family Physician were determinants of income inequality among GPs. Adjustment of income in terms of working hours indicated that one of the most important reasons for these differences is the workload of doctors in different groups. It also seems that factors such as different payment methods and a different insurance contract can explain these inequalities. Since the motivation and function of physicians can be influenced by income inequality, policy-makers in the health system should consider factors increasing such inequalities. For this purpose, further studies are needed to determine the factors affecting inequality and the extent to which each of these factors is effective.

### Study limitations

In this study, owing to lack of suitable access to GPs, the participants were selected through simple sampling. Therefore, the main limitation of this study is non-random sampling, which could overshadow the generalizability of the results.

## Additional file

Additional file 1: Checklist. A researcher-made checklist on the economic behavior of GPs. (DOCX 18 kb)

## Data Availability

The datasets used and analyzed during the current study are available from the corresponding author on reasonable request.

## Abbreviations

CME: Continuous Medical Education
GC: Gini coefficient
GPs: General practitioners
PPP: Purchasing Power Parity
UNDP: United Nations Development Programme
