# Supplementary material for: Income inequality among general practitioners in Iran: a decomposition approach
Source: BMC Health Serv Res. 2019 Sep 2;19:620. doi: 10.1186/s12913-019-4473-7 (PMC6721336; doi:10.1186/s12913-019-4473-7)
Supplement: Supplementary file 1 — Checklist. A researcher-made checklist on the economic behavior of GPs. (DOCX 18 kb) [file 12913_2019_4473_MOESM1_ESM.docx]

**Dear general practitioner**

The present checklist is developed to investigate economic performance of Iranian general practitioners. The research project is approved, sponsored, and supervised by Shiraz University of Medical Sciences under code [15918](http://pajooheshyar.sums.ac.ir/user/view_tarh_form.phtml?seed=7PNyWAEM6iYHEfyfFxlJBq9zd9i1zmjf3sJWaMwrWkVZWY2TVp&cod_tarh=1396-01-68-15918). Please complete the following questions. Your information will be kept confidential**.**

**Demographic information**

1- Birth Date: ………………

2- Gender: ………………...

Male ○

Female ○

3- Marital status:

Single ○

Married ○

**Practice information**

1 - Where do you practice?

Office ○

Public clinic ○

Private clinic ○

Public hospital ○

Private hospital ○

Other settings ○

2- Do you practice as a family physician?

Yes ○

No ○

3- Where is your practice location?

City ○ City name? ……….... Province name: …...…......

Village ○ Village name? ……….... Province name: ……....

4- How many years do you practice as a physician? …..

5- How much do you earn averagely from your medical practice before taxes per month? ………… Rials

6- How many hours do you work in a day? ……………

7- How many days do you work in a month? ………….
